# Characterization of the Placenta in the Newborn with Congenital Heart Disease: Distinctions Based on Type of Cardiac Malformation

**DOI:** 10.1007/s00246-018-1876-x

**Published:** 2018-05-04

**Authors:** Jack Rychik, Donna Goff, Eileen McKay, Antonio Mott, Zhiyun Tian, Daniel J. Licht, J. William Gaynor

**Affiliations:** 1 Fetal Heart Program, Children’s Hospital of Philadelphia, Philadelphia, USA; 2 Department of Pediatrics, Perelman School of Medicine at the University of Pennsylvania, Philadelphia, USA; 3 Division of Pediatric Cardiology, Loma Linda University Children’s Hospital, Loma Linda, USA; 4 Department of Pathology, Texas Children’s Hospital, Baylor College of Medicine, Houston, USA; 5 Division of Pediatric Cardiology, Department of Pediatrics, Texas Children’s Hospital, Baylor College of Medicine, Houston, USA; 6 Division of Neurology, Children’s Hospital of Philadelphia, Philadelphia, USA; 7 Department of Pediatrics, Perelman School of Medicine, University of Pennsylvania, Philadelphia, USA; 8 Division of Cardiothoracic Surgery, Children’s Hospital of Philadelphia, Philadelphia, USA; 9 Department of Surgery, Perelman School of Medicine, University of Pennsylvania, Philadelphia, USA; 10 Fetal Heart Program, Cardiac Center, Children’s Hospital of Philadelphia, 3401 Civic Center Boulevard, Philadelphia, PA 19066 USA

**Keywords:** Placenta, Fetal echocardiography, Congenital heart disease

## Abstract

The placenta is a complex organ that influences prenatal growth and development, and through fetal programming impacts postnatal health and well-being lifelong. Little information exists on placental pathology in the presence of congenital heart disease (CHD). Our objective is to characterize the placenta in CHD and investigate for distinctions based on type of malformation present. Placental pathology from singleton neonates prenatally diagnosed and delivered at > 37 weeks gestation was analyzed. Placental findings of absolute weight, placental weight-to-newborn birth weight ratio, chorangiosis, villus maturity, thrombosis, and infarction were recorded and analyzed based on four physiological categories of CHD: (1) single ventricle-aortic obstruction, (2) single ventricle-pulmonic obstruction, (3) two-ventricle anomalies, and (4) transposition of the great arteries (TGA). Associations between fetal Doppler assessments of middle cerebral/umbilical arterial flow and placental findings were investigated. A total of 120 cases of complex CHD were analyzed. Overall placental-to-birth weight ratios were < 10th percentile for 77% and < 3rd percentile for 49% with abnormalities of chorangiosis (18%), hypomature villi (15%), thrombosis (41%), and infarction (17%) common. There was no association between fetal Doppler flow measures and placental abnormalities. Newborns with TGA had the greatest degree of placental abnormality. Placentas of newborns with CHD are smaller than expected and manifest a number of vascular abnormalities, with TGA most prominent. Fetal Doppler does not correlate with these abnormalities. Studies investigating the relationship between placental abnormalities and postnatal outcomes may offer insight into the fetal origins of outcome variability in CHD.

## Introduction

The placenta is one of the least understood of human organs and arguably one of the most important, not only for the health of a woman and her fetus during pregnancy, but also for the lifelong health of both [1, 2]. The human placenta manifests a number of unique biological characteristics. It is a complex and highly structured organ with rich vascular contributions from two entities, mother and fetus. It is the only human organ than can be grown as new, discarded, then de novo grown again. The placenta is the interface between mother and fetus during the earliest period of human development, and thus plays a critical role in influencing the fundamental processes of organogenesis. Furthermore, epidemiological studies have demonstrated the important role of the placenta in “programming” human health well beyond the fetal and neonatal periods of life, a phenomenon now recognized as the fetal origins theory or the “Barker hypothesis” [3]. Through its influence in setting the stage for organ system structure and function at the very outset of life, the placenta and the in utero environment it creates influence the development of a wide range of disorders in later adult life including atherosclerotic disease, hypertension, cancer, and other chronic conditions [4, 5].

The role the placenta plays in congenital heart disease, the most common birth defect in the human, is poorly understood. Placentation and early fetal cardiovascular development may be linked in a number of ways and it is reasonable to suspect that abnormalities in one process may be associated with the other. In mothers carrying a fetus with congenital heart disease (CHD), an imbalance in pro-angiogenic and anti-angiogenic factors in both maternal blood and fetal cord blood is reported [6]. Maternal preeclampsia, a clinical consequence of placental vascular dysgenesis, is associated with fetal CHD, suggesting a shared pathway of development between these conditions, with an aberration in early angiogenesis possibly resulting in abnormality of both the placenta and the fetal cardiovascular system [7, 8]. Not only is it plausible to suspect that the placenta is de-novo abnormally formed in the fetus with CHD, but differences in blood flow patterns and perfusion characteristics unique to the type of heart malformation present may further influence growth and development of the placenta during gestation. For example, relatively low cardiac output or altered oxygen delivery as seen in conditions such as hypoplastic left heart syndrome [9] or transposition of the great arteries [10], respectively, may affect placental growth and development, further impacting functionality of this essential interface for fetal maturation.

Placental structure and function may influence outcomes in the fetus with CHD and is thus worthy of exploration. Investigational focus on the placenta might offer clues that can prognosticate for outcomes, as well as identify potentially modifiable variables that may prove to be candidates for prenatal therapy. In order to better understand the relationship between the placenta and CHD, the objectives of this study are to (1) investigate characteristics of the placenta at birth in CHD, (2) explore for differences in these characteristics based on type of heart malformation present, and (3) determine the possible relationship between placental characteristics and fetal echocardiographic Doppler parameters of blood flow in a variety of forms of CHD.

## Methods

This is a single center cohort study. Fetuses with CHD prenatally diagnosed and managed at the Fetal Heart Program at the Children’s Hospital of Philadelphia (CHOP) and delivered in our Special Delivery Unit at CHOP were enrolled. Subjects include (1) singleton fetuses with hemodynamically important congenital heart disease, defined as anomalies requiring surgical or catheter-based intervention within the first 6 months of life, and (2) fetuses delivered ≥ 37 weeks gestation and for whom an intact placenta was available for pathology examination. In order to reduce bias and diminish influence from confounding factors, subjects are excluded if there was any known identifiable genetic, chromosomal, or syndromic diagnosis before or after birth, any major additional anomaly (i.e., diaphragmatic hernia), or prematurity < 37 weeks gestation. Institutional Review Board permission was obtained for the study.

Placenta pathology reports were reviewed with tabulation of predetermined anatomical and pathological characteristics. Placental examinations were performed per standardized institutional protocol with initial review by any one of the three perinatal pathologists. Finalized reports and placental images were retrospectively reviewed by one pathologist (EM) for the following variables: placental weight, villous maturity, villous chorangiosis, villous infarction, and thrombosis. The diagnosis of chorangiosis is based on the previously defined histologic criteria of ≥ 10 villi containing ≥ 10 capillaries in ≥ 10 microscopic fields [11]. Villus maturity is determined by pathologist interpretation of overall villous appearance accounting for ratio of terminal to stem villi, villous vascularity including adequacy of vasculo-syncytial membrane formation, presence of cytotrophoblast, and presence of syncytial knots [12]. Thrombosis is defined as the presence of inter-villous thrombohematoma, sometimes but not always associated with placental infarction.

Records were reviewed for birth weight and then converted to *z* score for gestational age at birth [http://peditools.org/fenton2013/index.php] [13]. As there is no universal standardized consensus on placental weight measures, we used two commonly applied methods: (1) birth weight-to-placental weight ratio compared to normal for gestational age [14] and (2) placental weight-to-birth weight ratio, then reported as the number and percent of subjects with placental weight-to-birth weight ratio at < 10th percentile and at < 3rd percentile for gestational age for the normal population [15].

Subjects underwent fetal echocardiography per our laboratory clinical protocol [16] including Doppler echocardiography of the umbilical artery (UA) and middle cerebral artery (MCA) [17], with calculation of the pulsatility index (PI) as a measure of vascular impedance, with PI = [peak systolic velocity minus end diastolic velocity] divided by the mean velocity. Data from the last fetal echocardiogram study prior to birth were reviewed. Three waveforms were measured and the average taken for analysis. The ratio of MCA PI-to-UA PI was calculated as the cerebro-placental resistance ratio (CPR), an index of the circulatory resistance ratio of the cerebrovasculature-to-placental vasculature on the fetal side [18]. A healthy normal CPR is > 1, indicating a high cerebrovascular resistance relative to a low placental vascular resistance [19].

Data are reported for the entire cohort and comparisons made between subsets based on categories of CHD. In order to detect for possible differences in placental characteristics specific to type of congenital heart disease, subjects were divided into categories based on type of cardiovascular physiology as follows: (1) single ventricle with aortic obstruction (group 1), (2) single ventricle with pulmonic obstruction (group 2), (3) two-ventricle anatomy (group 3), and (4) transposition of the great arteries with no, or hemodynamically insignificant small, ventricular septal defect (group 4). Differences between groups for numerical values of birth weight, birth weight *z* scores, placental weight or weight ratios, and fetal Doppler flow indices are examined by one-way ANOVA. Differences between groups in the proportion of subjects with placental weight–birth weight ratios < 10%, or < 3% or with placental findings of thrombosis, infarction, chorangiosis, or hypomature villi are examined by Chi-square contingency table analysis. Associations between placental weight measures, thrombosis, infarction, villus maturity, and fetal Doppler blood flow indices are examined by regression analysis. *p* value of < 0.05 is considered significant.

## Results

One hundred and twenty subjects are included in the study. The distribution of anatomical type of CHD among the groups of physiological categories is listed in Table 1. Table 2 lists the newborn, placental, and fetal Doppler blood flow characteristics for all 120 subjects as well as for each of the groups.

**Table 1:** Spectrum of CHD

Group	Type of congenital heart anomaly	*N*
Group 1 = single ventricle with aortic obstruction (*n* = 33)	Hypoplastic left heart syndrome	28
	Double inlet left ventricle with coarctation/aortic arch hypoplasia	5
Group 2 = single ventricle with pulmonic obstruction (*n* = 21)	Complex single ventricle with severe pulmonary stenosis or pulmonary atresia	12
	Pulmonary atresia with intact ventricular septum, severe RV hypoplasia	9
Group 3 = two-ventricle anomalies (*n* = 51)	Tetralogy of Fallot	17
	Coarctation of aorta/aortic arch hypoplasia, with intact ventricular septum	7
	Coarctation of aorta/aortic arch hypoplasia with ventricular septal defect	6
	Truncus arteriosus (type I or II)	5
	Pulmonic stenosis, good size RV	5
	Aortic stenosis, good size LV	3
	Double-outlet right ventricle with pulmonic obstruction or no obstruction	3
	Complete common atrioventricular canal defect (balanced)	2
	Ebstein’s anomaly, pulmonic stenosis	1
	Interrupted aortic arch with ventricular septal defect	1
	Aortopulmonary window	1
Group 4 = transposition of the great arteries (*n* = 15)	Transposition of the great arteries, hemodynamically insignificant or no VSD, no outflow obstruction	15
Total	All anomalies	120

**Table 2:** Data by diagnosis of type of CHD

*n*	Total overall	Group 1 [1V A-obstr]	Group 2 [1V P-obstr]	Group 3 [2V]	Group 4 [TGA]	*p* Value
	120	33	21	51	15	
GA @ delivery (weeks)	38.9 ± 0.9	38.9 ± 0.84	39.1 ± 1.0	38.8 ± 0.86	39.2 ± 0.6	NS
Birth weight (g)	3220 ± 475	3227 ± 464	3274 ± 403	3132 ± 511	3428 ± 379	NS
Birth weight *z* score	− 0.02 ± 0.91	− 0.02 ± 0.93	0.03 ± 0.85	− 0.15 ± 0.89	0.37 ± 0.88	NS
Placental characteristics						
Placental weight (g)	449 ± 109	461 ± 99	489 ± 117	436 ± 113	428 ± 87	NS
B wt/P wt	7.42 ± 1.34	7.31 + 1.14	6.93 + 1.17	7.47 ± 1.43	8.23 + 1.32	< 0.05
P wt/ B wt ratio	0.14 ± 0.03	0.14 ± 0.02	0.15 ± 0.03	0.14 ± 0.03	0.12 ± 0.02	< 0.05
P wt/B wt ratio < 3%	59/120 (49%)	17/33 (52%)	8/21 (38%)	22/51 (43%)	12/15 (80%)	0.06
P wt/B wt ratio < 10%	93/120 (77%)	26/33 (79%)	18/21 (86%)	34/51 (67%)	15/15 (100%)	0.07
Thrombosis	43/105 (41%)	13/29 (45%)	7/19 (37%)	16/42 (38%)	7/15 (47%)	NS
Infarction	18/105 (17%)	6/29 (21%)	5/19 (26%)	5/42 (12%)	2/15 (13%)	NS
Chorangiosis	19/105 (18%)	4/29 (14%)	3/19 (16%)	7/42 (17%)	5/15 (33%)	0.09
Villus: hypomature	14/92 (15%)	4/26 (15%)	2/19 (11%)	3/34 (10%)	5/13 (38%)	0.07
Fetal Doppler characteristics						
GA @ fetal echo (weeks)	36.6 ± 1.6	37.1 ± 1.9	36.8 ± 1.3	36.2 ± 1.57	37.1 ± 1.08	NS
MCA PI	1.73 ± 0.46	1.47 ± 0.32	1.90 ± 0.37	1.86 ± 0.5	1.67 ± 0.38	< 0.001
UA PI	1.08 ± 0.24	1.09 ± 0.19	1.03 ± 0.22	1.11 ± 0.26	1.00 ± 0.30	NS
CPR	1.68 ± 0.58	1.38 ± 0.32	1.89 ± 0.61	1.77 ± 0.65	1.79 ± 0.48	< 0.005

**Fig. 1:**
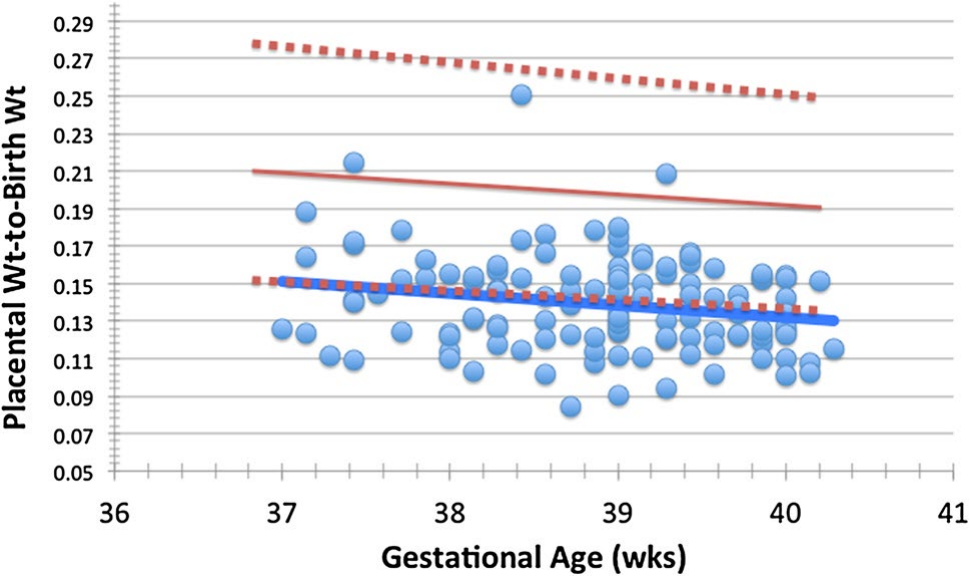
Display of the placental weight-to-birth weight ratio versus gestational age for 120 newborns with congenital heart disease. The solid red line represents the normal expected mean and the dotted red lines the 95% upper and lower confidence limits (as per Almog et al. [15]). The solid blue line represents the mean for the 120 study subjects. Note that, the mean line for the CHD group is nearly the same as the line for the lower 95% confidence limit for normal

**Fig. 2:**
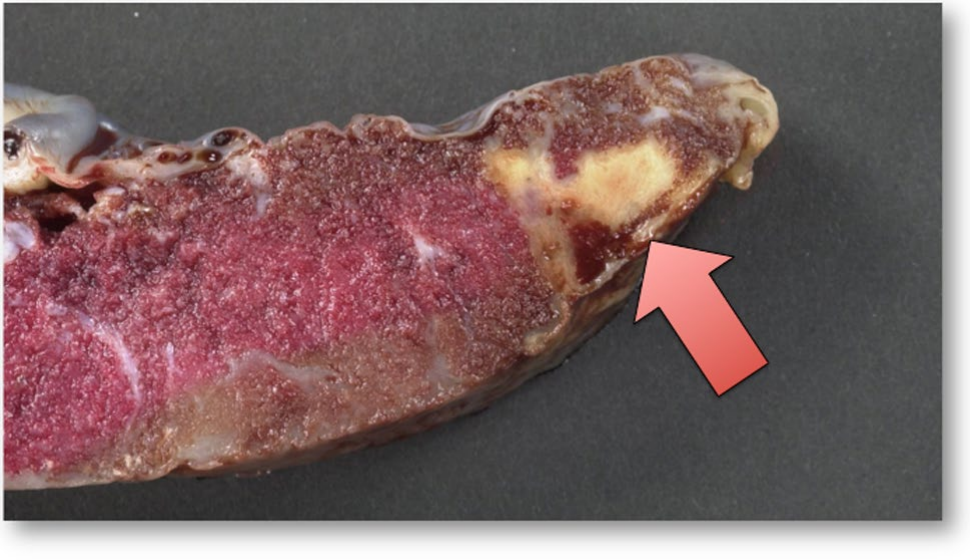
Placenta from a newborn with hypoplastic left heart syndrome. Arrow points to an area of infarction in the peripheral “vascular watershed” region of the placenta

Overall for the entire cohort, mean gestational age is 39 ± 0.9 weeks and birth weight *z* score is normal (− 0.02 ± 0.91); however, the placentas are smaller than expected. Overall, placental weight as characterized by birth weight-to-placental weight ratio is a mean of 7.42 ± 1.3, in comparison to expected for normal at a mean of 39 weeks gestational age of 7.1 as per Baergen [14]. The distribution of placental weight-to-birth weight ratio versus gestational age in comparison to expected for normal is depicted in Fig. 1. The mean placental weight-to-birth weight ratio for the normal population at a gestational age of 39 weeks is 0.20 ± 0.03. Nearly one-half of subjects have a placental weight-to-birth weight ratio of < 3rd percentile, and over three quarters of subjects have placental weight-to-birth weight ratio < 10th percentile (Table 1). Placental thrombosis is evident in 41% of cases, with placental infarction seen in 17% (Fig. 2). Abnormality of placental development, characterized as the presence of chorangiosis is noted in 18% and placental villous hypomaturity in 15%.

No significant differences in gestational age at delivery, birth weight or birth weight *z* score are noted between the different categories of CHD. Differences in the ratios of newborn birth weight to placental weight are present between the groups, with the highest ratio seen in TGA (group 4, *p* < 0.05). Differences between the groups in the proportion of subjects with placental weight-to-birth weight ratio at < 3rd percentile and at < 10th percentile for gestational age trended towards, but did not reach, statistical significance (*p* = 0.06, *p* = 0.07, respectively). TGA (group 4) has the greatest proportion of subjects with placental weight-to-birth weight ratios at < 3rd and < 10th percentile (80 and 100%, respectively). There are no differences between the groups in proportion of subjects with placental thrombosis or infarction. Difference between the groups in subjects with chorangiosis or hypomaturity of placental villi trended towards significance (*p* < 0.1) with the greatest proportion seen in TGA (group 4).

Fetal Doppler echocardiography was performed at a mean of 36.6 ± 1.6 weeks gestation for the entire cohort, with no differences between the groups. There is no difference in UA PI between the groups; however, there is a significant difference between the groups for MCA PI (*p* < 0.001) and for the derived calculated CPR (*p* < 0.005). The lowest MCA PI and CPR values are in group 1 (SV with aortic obstruction) and the highest are in group 2 (SV with pulmonic obstruction). There are no significant associations found between the placental parameters of weight, thrombosis, infarction, chorangiosis or villus hypomaturity and the fetal Doppler derived indices of blood flow to the UA or MCA.

## Discussion

Modern day surgical correction of congenital heart disease leads to improvement in hemodynamics in most cases, yet outcomes and quality of life for many survivors is limited. For example, the neonatal arterial switch operation for TGA leads to resumption of a normal cardiovascular state in the newborn; however, neurodevelopmental outcomes are impaired in a substantial proportion of children and adolescents [20, 21]. One explanation is that this may be due to alteration in organ development and newborn biological substrate, related to factors that are present prior to birth and are not modified by cardiac surgery. Fetal “programming” in CHD may be taking place within an altered maternal-fetal environment, in which the placenta plays a key role.

Despite its importance in influencing fetal and postnatal life, there is a relative paucity of knowledge concerning the structure and function of the placenta in congenital heart malformations. Based on a large population-based study from Denmark, lower placental weight at birth is associated with tetralogy of Fallot, double-outlet right ventricle and “major” ventricular septal defects [22]. In a small series of 16 subjects with hypoplastic left heart syndrome, the placenta is reported smaller than normal in weight with a marked increased in fibrin deposition, decreased terminal villi and increased expression of leptin, an angiogenic and mitogenic hormone produced by the placenta, which may indicate an attempt to compensate for vascular abnormalities [23]. Placental dysfunction-related complications such as preeclampsia are associated with CHD [7, 8]. Umbilical cord insertion abnormalities of eccentric, marginal or velamentous cord insertions are also noted with increased frequency in CHD [24]. These findings suggest the concept of an early, common origin to both abnormal placentation and developmental abnormalities of the fetal cardiovascular system.

In our study, we found abnormally low placental weight-to-birth weight ratios for newborns with CHD as well as abnormalities of placental thrombosis, infarction, chorangiosis, and hypomature villi. When present, thrombosis and infarction were most notable in the periphery of the placenta in “watershed” regions in which the circulation may be most vulnerable to compromise. Chorangiosis is of particular interest as it indicates an increase in capillary density per region of villous tissue and is proposed to reflect long-standing, low-grade placental hypoxia [25]. Chorangiosis is reported in a number of clinical complications of pregnancy such as maternal diabetes and preeclampsia and is associated with increased risk of placental thrombosis [26]. Chorangiosis is present in nearly 1 out of 5 overall in our cohort, with placental thrombosis in 2 out of 5. It is conceivable that selected fetuses with CHD may exhibit a thrombotic placental vasculopathy, and if so, these may benefit from anti-thrombotic therapies like aspirin, as is commonly used in other conditions of high-risk pregnancies [27].

In order to better understand the possible influence of fetal cardiovascular physiology on placental characteristics at birth, we analyzed our findings based on physiological categories of CHD. Although placental abnormalities are present in all physiological groups, our data provide a “signal” for one type in particular, with the most profound differences noted in TGA. Newborns with TGA have the smallest placenta relative to birth weight with all TGA subjects in our study falling below the 10th percentile. In addition, those with TGA also had the highest frequency of chorangiosis and villi hypomaturity (present in 1/3, twice as common in comparison to the other categories) potentially reflecting placental vascular derangement.

Why are these findings present to a greater degree in newborns with TGA? It is possible that there may be a primary early co-incident derangement in placental formation occurring very early in gestation at the same time as a defect in septation of the great arteries from the common arterial trunk. Alternatively, the patterns of fetal blood flow unique to TGA may be a contributor. In the fetus with TGA, umbilical arterial oxygen delivery to the placenta is lower than normal. Streaming of highly oxygenated blood exiting the placenta through the umbilical vein is directed via the ductus venosus to the left side of the heart, which is connected to the pulmonary artery, while poorly oxygenated blood is directed towards the right side of the heart, which is connected to the aorta. Streaming phenomena and mixing result in blood oxygen content in the descending aorta and the umbilical arteries that is lower than normal, and thus oxygen delivery to the developing placenta throughout gestation may be reduced. Novel fetal MRI techniques are now able to quantify blood oxygen content in utero and have recently confirmed in vivo low arterial saturation in various forms of CHD [10]. These investigators also discovered low umbilical venous saturation levels in cases of CHD suggesting dysfunction of the placenta in its role as the organ of fetal oxygenation. Depending upon the type of cardiac structural abnormality present, blood oxygen content *to*, as well as blood oxygen content exiting *from* the placenta, may be different than normal.

While our newborns with TGA were relatively larger in weight, their placentas were the smallest in absolute weight size compared to the other physiological categories, although neither newborn nor placental weight alone reached statistical significance. Nevertheless, the ratio of birth weight-to-placental weight was the highest for any of our physiological categories. This raises some provocative questions: are fetuses with TGA more “efficient” at utilization of placental tissue, with good somatic growth taking place with a smaller placenta? Is it possible that somatic growth in TGA is taking place at the expense of neural maturation and growth, in the presence of limited placental mass? Although conjecture at this time, it is worth considering the possibility that the neurodevelopmental delay seen in TGA and other forms of CHD such as hypoplastic left heart syndrome may in part have its origins in fetal life due to placental abnormalities such as small placental size, poor vascularity or thrombosis [28]. Further study looking at placental characteristics in-vivo during gestation with longitudinal follow-up and clinical outcomes is necessary in order to answer these questions.

We found no relationship between placental characteristics and fetal echocardiography Doppler derived flow resistance indices of the umbilical artery, or as ratios of middle cerebral artery-to-umbilical artery. As anticipated there is an association between type of CHD and distinctions in middle cerebral artery pulsatility indices, as previously reported [29]. These likely reflect local changes in cerebrovascular impedance based on cardiac blood flow patterns, with the lowest middle cerebral artery impedance noted in those with single ventricle and aortic obstruction (e.g., hypoplastic left heart syndrome). The condition of fetal growth restriction when no CHD is present is typically due to placental “insufficiency,” with elevated placental vascular resistance manifesting as a high umbilical artery Doppler pulsatility index. The fact that we found no association between umbilical artery Doppler data and any of our placental pathology findings suggests a different mechanism of placental pathophysiology in the fetus with CHD compared to the fetus with classic growth restriction. For example, chorangiosis—an increase in capillary density per placental tissue—may in fact be a compensatory response that may *decrease* overall placental vascular resistance by increasing the vascular cross-sectional area. Fetal echocardiography-derived Doppler flow measures such as pulsatility index calculations are a measure of vascular resistance, not flow. Doppler-derived volumetric calculations of umbilical venous flow as an absolute measure of placental blood flow, or perhaps as a relative fraction of overall fetal combined cardiac output may be more informative [30]. Other methodologies such as MRI techniques will add to the capacity to better gauge the relationship between blood flow and placental structure and function during pregnancy, which will complement fetal echocardiography and add important insights to our understanding [10, 31].

Our study is limited in its conclusions in large part by the absence of postnatal outcome data to correlate with our placental findings. Nevertheless, our findings of placental abnormality are hypothesis generating as we look to identify worthy elements to incorporate into larger longitudinal studies. We report on static postnatal placental observations at birth, and do not provide information on the temporal progression of these findings during pregnancy. Development of safe and non-invasive surveillance techniques for characterizing the human placenta in a longitudinal manner from the earliest point of first trimester and onward is necessary and is currently the focus of a multicenter effort under the NIH sponsored Human Placenta Project [http://nichd.nih.gov/hpp] [2]. Furthermore, our study is limited by the absence of direct comparison to a contemporaneous normal population analyzed in the same manner as the CHD group. A wide spectrum of placental changes and weights can be seen in the healthy newborn population, thereby limiting the conclusions that can be made from the prevalence of findings we identify.

Our findings support the value of further investigation of the placenta in CHD, in particular in patients with TGA. Prospective investigation looking at details of maternal factors influencing placental health (i.e., overall health, nutrition, preeclampsia, diabetes) will be important to incorporate into future study analysis. Knowledge gained may identify modifiable variables during pregnancy leading to treatment such as maternal supplemental oxygenation, or maternal anti-thrombotic therapy in select pregnant women, thus ushering in an era of fetal therapy that may improve outcomes for patients with CHD.
